# HIV-1 transmitted drug resistance-associated mutations and mutation co-variation in HIV-1 treatment-naïve MSM from 2011 to 2013 in Beijing, China

**DOI:** 10.1186/s12879-014-0689-7

**Published:** 2014-12-16

**Authors:** Yang Jiao, Shuming Li, Zhenpeng Li, Zheng Zhang, Jianhong Zhao, Li Li, Lijuan Wang, Qianqian Yin, Yan Wang, Zhaoli Zeng, Yiming Shao, Liying Ma

**Affiliations:** State Key Laboratory for Infection Disease Prevention and Control, National Center for AIDS/STD Control and Prevention (NCAIDS), Collaborative Innovation Center for Diagnosis and Treatment of Infectious Diseases, Chinese Center for Disease Control and Prevention (China CDC), Beijing, 102206 China; Beijing Chaoyang District Centre for Disease Control and Prevention, Beijing, 100021 China

**Keywords:** HIV-1, MSM, Subtypes, Transmitted drug resistance-associated mutations, Co-variation

## Abstract

**Background:**

Transmitted drug resistance (TDR) is an important public health issue, because TDR-associated mutation may affect the outcome of antiretroviral treatment potentially or directly. Men who have sex with men (MSM) constitute a major risk group for HIV transmission. However, current reports are scarce on HIV TDR-associated mutations and their co-variation among MSM.

**Methods:**

Blood samples from 262 newly diagnosed HIV-positive, antiretroviral therapy (ART)-naïve MSM, were collected from January 2011 and December 2013 in Beijing. The polymerase viral genes were sequenced to explore TDR-associated mutations and mutation co-variation.

**Results:**

A total of 223 samples were sequenced and analyzed. Among them, HIV-1 CRF01_AE are accounted for 60.5%, followed by CRF07_BC (27.8%), subtype B (9.9%), and others. Fifty-seven samples had at least one TDR-associated mutation, mainly including L10I/V (6.3%), A71L/T/V (6.3%), V179D/E (5.4%), and V106I (2.7%), with different distributions of TDR-associated mutations by different HIV-1 subtypes and by each year. Moreover, eight significant co-variation pairs were found between TDR-associated mutations (V179D/E) and seven overlapping polymorphisms in subtype CRF01_AE.

**Conclusions:**

To date, this work consists the most comprehensive genetic characterization of HIV-1 TDR-associated mutations prevalent among MSM. It provides important information for understanding TDR and viral evolution among Chinese MSM, a population currently at particularly high risk of HIV transmission.

**Electronic supplementary material:**

The online version of this article (doi:10.1186/s12879-014-0689-7) contains supplementary material, which is available to authorized users.

## Background

The increasing availability of antiretroviral therapy (ART) worldwide has significantly reduced mortality and improved quality of life for HIV-infected patients. However, the therapeutic effect of ART is weakened by the emergence of drug-resistant mutant viruses. Transmission of drug-resistant (TDR) strains to persons who are ART-naïve can compromise the effectiveness of treatment and limit antiretroviral regimens options. TDR consists 10 ~ 20% of new HIV-1 infections worldwide [1]. In resource-limited countries such as China, where ART is provided to patients for free through the “Four Free One Care” policy [2], TDR against standard ART regimens threatens the stability of treatment programs and must be carefully monitored.

Several factors contribute to the occurrence of TDR, including frequency of exposure to non-treatment naïve viruses, ART regimen efficacy in the transmitting patient, rates of virologic suppression, and genetic diversity and replicative capacity of the viral strains in question. To our interest, evidence suggests that HIV-1 genetic diversity may influence the type and rate of resistance mutations that may eventually emerge upon drug exposure [3],[4]. Previous studies showed a significant correlation between treatment-associated mutations and overlapping polymorphisms in the RT and PR viral genes [5]. We postulate that there may be co-variation between TDR-associated mutations and overlapping polymorphisms on treatment-naïve patients, which affect the transmission of drug resistance mutant viruses.

Previous evidence suggests that drug resistance mutations may result in significantly decreased replicative fitness and hence transmission efficacy [6]. Thus, we may expect persons at higher frequency of exposure to non-treatment-naïve viruses to be at higher risk of receiving drug resistant strains than those persons under lower frequency of exposure. In China, one such risk group with particularly high frequency of HIV-1 exposure is men who have sex with men (MSM). MSM in China typically have multiple sexual partners, low rates of condom usage, and low rates of HIV screening [7],[8]. As well, they are a rapidly expanding high risk population for HIV transmission: the proportion of all reported cases of HIV infection in China with history of MSM sex has increased from 2.5% in 2006 to 13.7% in 2011 [9]. It is therefore important to examine TDR among MSM in China.

Our study focuses on Beijing, where the proportion of MSM carrying HIV has increased rapidly from 3.1% in 2002 [10] to 4.8% in 2006 [11]. The proportion of MSM among newly HIV diagnosed cases was 70.7% in 2012 [12], much higher than the corresponding rates in other cities in China [13]-[15]. Although several studies have reported TDR among ART-naïve MSM in Beijing [11],[16],[17], there lacks a systematic analysis on TDR rates over time and the co-variation of TDR-associated mutations. Thus, we performed a comprehensive genetic characterization of HIV-1 strains prevalent in MSM in Beijing from 2011 to 2013, analyzing TDR-associated mutations and mutation co-variation.

## Methods

### Study patients

A total of 262 HIV-1 positive individuals were randomly recruited from 2011 to 2013 at voluntary counseling and testing sites (VCT) in Beijing Chaoyang District Center for Disease Control and Prevention, following three criteria: having had history of MSM sex, being ART-naïve and newly diagnosed. This study was approved by the Institutional Research Ethics Community, China Chaoyang CDC, and all subjects signed informed consent forms prior to blood collection. Epidemiological data was collected by trained interviewers. HIV-1 infection status was determined by an enzyme immunoassay (ELISA, Wantai, China) and confirmed by Western blot assay (HIV BLOT 2.2, MP Diagnostics, Singapore). Blood plasma was separated and stored at −70°C prior to genetic analysis.

### HIV-1 RNA extraction, amplification and sequencing

Viral RNA was extracted from 200 μl EDTA-anticoagulated plasma using a QIAamp viral RNA kit (Qiagen Inc., Germany) according to the manufacturer’s instructions. The HIV-1 pol gene (1,197 bp length), containing the full-length protease (PR) gene and the first 300 codons of the reverse transcriptase (RT) gene, were amplified and sequenced, using an in-house drug resistance genotyping method as previously described [18]. The target sequence was amplified with One Step Reverse Transcription PCR reagents (Qiagen Inc., Germany) using primers listed in Table 1. Amplification steps were as follows: reverse transcription at 50°C for 30 min, pre-denaturation at 94°C for 5 min, 30 cycles of denaturation at 94°C for 30 s, annealing at 55°C for 30 s, extension at 72°C for 2.5 min, and an additional extension at 72°C for 10 min. Nested PCR was performed using Taq PCRmaster mix (Qiagen Inc., Germany) with primers in Table 1. The cycling conditions were: pre-denaturation at 94°C for 5 min, 30 cycles of denaturation at 94°C for 30 s, annealing at 63°C for 30 s, extension at 72°C 2.5 min, and an additional extension at 72°C for 10 min. PCR products were visualized by 1% agarose gel electrophoresis and sequenced using ABI 3730xl Automated DNA Analyzer (Applied Biosystems, Foster City, CA). Each step was carried out with negative controls.

**Table 1:** **Primers used in the optimized in-house assay**

Primer name	Sequence (5,-3,)	Location (based on HXB2)	Purpose
MAW 26	TTGGAAATGTGGAAAGGAAGGAC	2028-2050	RT-PCR
RT21	CTGTATTTCTGCTATTAAGTCTTTTGATGGG	3509-3539	RT-PCR
PRO-1	CAGAGCCAACAGCCCCACCA	2147-2166	Nested PCR
RT20	CTGCCAGTTCTAGCTCTGCTTC	3441-3462	Nested PCR
MAW26-07BC	TGGAAATGTGGAAAAGAAGGAC	2028-2050	RT-PCR
RT21-07BC	CTGTATTTCAGCTATCAAGTCTTTTGATGGG	3509-3539	RT-PCR
PRO1-07BC	CAGAGCCAACAGCCCCACCA	2147-2166	Nested PCR
RT20-07BC	CTGCCAATTCTAATTCTGCTTC	3441-3462	Nested PCR
MAW26-01AE	TGGAAATGTGGRAARGAAGGAC	2028-2050	RT-PCR
RT21-01AE	GTAYTTCTGCTAYTAAGTCTTTTGATGGG	3511-3539	RT-PCR
PRO1-01AE	CAGAGCCAWCAGCCCCACCA	2147-2166	Nested PCR
RT20-01AE	CTGCCAAYTCTAATTCTGCTTC	3441-3462	Nested PCR

### Phylogenetic analysis

All assembled sequences were submitted to the Los Alamos National Laboratory HIV Sequence Database (http://www.hiv.lanl.gov/content/index) to determine HIV genotype, which were further confirmed by phylogenetic analysis using standard reference sequences representing subtypes A–D, F–H, J, K, CRF01_AE, CRF07_BC, and CRF08_BC (www.hiv.lanl.gov). DNA alignment was performed by the Clustal W method using MEGA5 [19], followed by manual adjustment. Phylogenetic analysis was also conducted with MEGA5 using neighbor-joining trees under a Kimura 2-parameter model and tested by the bootstrap method with 1,000 replicates.

### Drug resistance analysis

Sample pol gene sequences were compared to a consensus sequence using HIV db software (Stanford HIV Drug Resistance Database, http://hivdb.stanford.edu, version 7.0) to detect drug resistance mutations, including major and minor protease inhibitor (PI) resistance mutations, nucleoside reverse transcriptase inhibitor (NRTI), and non-nucleoside reverse transcriptase inhibitor (NNRTI) resistance mutations.

### Co-variation analysis between TDR-associated mutations and positively selected mutations

We analyzed co-variation between TDR-associated mutations and positive selected mutation using the CorMut package [20]. Briefly, the procedure was: positively selected mutations were identified using selection pressure (Ka/Ks ratio) based method [21],[22], in which a Ka/Ks value of >1 indicates a positive selection. Log odds (LOD) confidence score was used to measure the significance of selection pressure (cut off > = 2). The 05GX001 strain (subtype CRF01_AE) was used as a reference when performing the computation. The Jaccard similarity coefficient was used to measure the co-variation between TDR mutations and positively selected mutations. Fisher’s exact test was used to check the significance of co-variation. False discovery rate was controlled using the Benjamini and Hochberg procedure with a 0.2 cut-off. P adjusted value < 0.2 was considered statistical significance.

## Results

### Patient characteristics

Among 262 HIV-1 positive samples, the pol genes of 223 samples (85.11%) were successfully amplified and sequenced. The mean age of the 223 patients was 30.3 (range: 17–64). 75.3% of subjects were never married, 21.5% were married, and 3.1% were divorced or widowed. More than two-thirds of participants (69.5%) had received college-level or higher education degree. The basic demographic characteristics are shown in Table 2.

**Table 2:** **Characteristic and genotypes of study subjects**

	Total	Year		
		2011	2012	2013
Total	223	21	126	76
Subtype				
CRF01_AE	135(60.53%)	12(57.14%)	80(63.49%)	43(56.58%)
CRF07_BC	62(27.80%)	7(33.33%)	31(24.60%)	24(31.58%)
B	22(9.86%)	2(9.53%)	12(9.52%)	8(10.53%)
Others	4(1.79%)	0	3(2.38%)	1(1.32%)
Age				
Mean	30.79	27.76	31.35	30.71
≤24	48(21.52%)	7(33.33%)	23(18.26%)	18(23.68%)
25-34	116(52.02%)	11(52.38%)	71(56.35%)	34(44.74%)
35-44	44(19.73%)	3(14.29%)	21(16.67%)	20(26.32%)
≥45	15(6.73%)	0	11(8.73%)	4(5.26%)
Marriage status				
Single	168(75.34%)	16(76.19%)	93(73.81%)	59(77.63%)
Married	48(21.52%)	4(19.05%)	30(23.81%)	14(18.42%)
Divorced/widowed	7(3.14%)	1(4.76)	3(2.38%)	3(3.95%)
Education				
Middle school and below	26(11.66%)	3(14.29%)	15(11.90%)	8(10.53%)
High school	42(18.83%)	4(19.05%)	28(22.22%)	10(13.16%)
College level and above	155(69.51%)	14(66.67%)	83(65.87%)	58(76.32%)

### HIV-1 genetic characteristics

**Figure 1:**
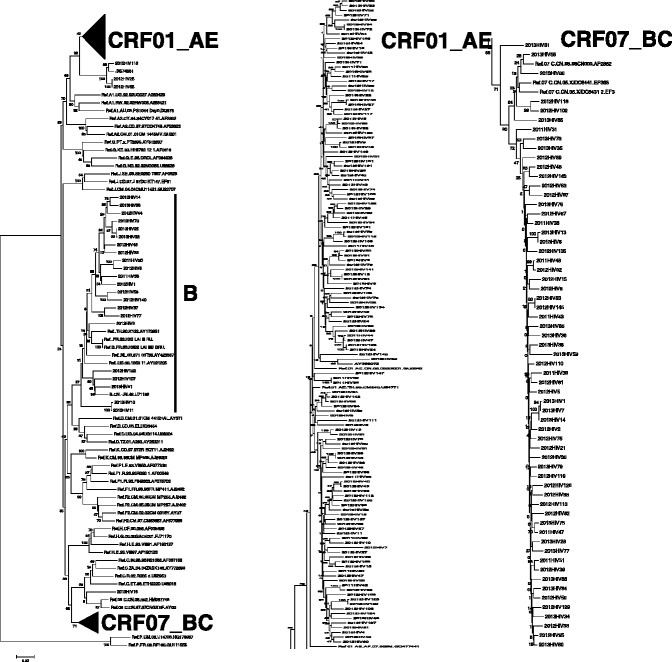
**Phylogenetic trees of HIV-1 pol genes were constructed using MEGA 5 based on neighbour-joining methods.** The samples’ sequences and reference HIV-1 subtypes (subtypes A–D, F–H, J, K, CRF01_AE, CRF07_BC, CRF08_BC, and group O) available in the Los Alamos database were aligned using CLUSTAL W with minor manual adjustments. The statistical robustness of the neighbour-joining tree and reliability of the branching patterns were confirmed by bootstrapping (1000 replicates). The trees were midpoint rooted. Values on the branches represent the percentage of 500 bootstrap replicates.

Phylogenetic analysis of the amplified pol gene regions (1197 bp) showed that the samples were generally tightly clustered within their respective subtypes (Figure 1). Their genotype distribution was as follows: 135 cases (60.5%) were subtype CRF01_AE, 62 cases (27.8%) were subtype CRF07_BC, 22 cases (9.9%) were subtype B, two were CRF01B, one was CRF55_01B, and one was CRF08_BC (Table 2). There was no significant difference in HIV-1 subtype distributions between each year.

### Prevalence of transmitted drug resistant variants

Amplified gene regions were assessed for TDR-associated mutations through the Stanford HIV Drug Resistance Database. Mutations were classified and summarized according to their ability of conferring resistance to PI, NRTI, or NNRTI drug classes. Among our samples, 25.6% (57/223) had TDR-associated mutations, including 16.1% (36/223) for PI mutations, 0.9% (2/223) for NRTI mutations, and 9.0% (20/223) for NNRTI mutations. PI mutations included L10I/V (7.2%, 16/223) and A71L/T/V (6.3%, 14/223), with relatively high frequency; NRTI mutations, included L74I (0.45%, 1/223) and V75L (0.45%, 1/223); and the most frequent NNRTI mutation was V179D/E (5.4%, 12/223). Among these detected mutations, only L74I, M46L, G190E and E138G may result in drug resistance directly. Most of these mutations only conferred potential drug resistance. The detailed frequency of the mutations was shown in Table 3. Only six (2.7%) samples carried mutations conferring known levels of drug resistance, with 1.35% (3/223) against PIs (nelfinavir), 0.45% (1/223) against NRTIs (abacavir, didanosine), and 0.90% (2/223) against NNRTIs (efavirenz, etravirine, nevirapine, riplivirine).

**Table 3:** **HIV TDR-associated mutations identified among different subtypes/year in treatment-naïve Beijing MSM**

	PI	PI mutations	NRTI	NRTI mutations	NNRTI	NNRTI mutations	Total
Subtype							
CRF01_AE	14.07% (19/135)	L10I/V(14/19) V11I(2/19) L33F(2/19) M46L(2/19) A71L(1/19)	1.48% (2/135)	L74I(1/2) V75L(1/2)	8.15% (11/135)	V106I(1/11) V179D/E(9/11) G190E(1/11)	23.70% (32/135)
CRF07_BC	19.35% (12/62)	L10I(1/12) L33I(1/12) Q58E(2/12) A71T/V(8/12)	0	—	1.61% (1/62)	E138G(1/1)	20.97% (13/62)
Subtype B	22.72% (5/22)	L10I(1/5) A71T/V(5/5)	0	—	31.81% (7/22)	V106I(5/7) , V179E(2/7)	54.54% (12/22)
Year							
2011	4.77% (1/21)	L10I(1/1)	0	—	9.52% (2/21)	V106I(2/2)	14.29% (3/21)
2012	17.24% (22/126)	L10I/V(8/22) V11I(2/22) L33I/F(3/22) M46L(2/22) Q58E(1/22) A71T/V(6/22)	2.63% (2/126)	L74I(1/2),V75L(1/2)	11.1% (14/126)	V106I(4/14) E138G(1/14) V179D/E(8/14) G190E(1/14)	29.37% (37/126)
2013	17.11% (13/76)	L10I/V(5/13) Q58E(1/13) A71T/V(8/22)	0	—	5.26% (4/76)	V179D(4/4)	22.37% (17/76)
Total	16.14% (36/223)		0.89% (2/223)		8.52% (19/223)		25.56% (57/223)

PI, protease inhibitor resistance mutation; NRTI, nucleoside reverse transcriptase inhibitor resistance mutation; NNRTI, non-nucleoside reverse transcriptase inhibitor resistance mutation.

The type and frequency of TDR-associated mutations were different among different HIV-1 subtypes. The proportion of TDR-associated mutations was 23.7% (32/135) among CRF01_AE recombinant strains, the most frequent mutations being L10I/V and V179D/E. Among CRF07_BC recombinant strains, 21.0% (13/62) had TDR-associated mutations, A71T/V being the most frequent. Among subtype B, 54.5% (12/22) had TDR-associated mutations, A71T/V and V106I being the most frequent. Detailed information is summarized in Table 3.

Of note, the distribution of TDR-associated mutations differed by sampling year (Table 3). In 2011, 14.2% (3/21) had TDR-associated mutations, while the latter two years saw rates that were more than two times as high, with 29.4% (37/126) in 2012 and 22.4% (17/76) in 2013.

### Co-variation between transmitted drug resistance and polymorphisms

Co-variation analysis was performed to determine mutations or polymorphisms that were positively selected in association with TDR-associated mutations (Table 4). Eight mutation pairs with significant co-variation were identified in the RT region for CRF01_AE strains between the V179D/E TDR-associated mutation and seven overlapping polymorphisms. No significant mutation pair was identified for the PR region and for other subtypes.

**Table 4:** **Co-variation pairs between overlapping polymorphisms and TDR-associated mutations**

TDR-associated mutations	Polymorphisms	Jaccard index	P value	P value (adjusted)
V179D	R238K	0.19047619	0.003425851	0.052072937
V179D	A272P	0.125	0.020837895	0.197960003
V179E	T11K	0.07894737	0.020123086	0.197960003
V179E	I173K	0.08333333	0.01703163	0.197960003
V179E	K174Q	0.14285714	0.003172559	0.052072937
V179E	S207Q	1	2.39E-06	0.000181289
V179E	S211K	0.42857143	8.35E-05	0.003172559
V179E	R238K	0.15789474	0.002311436	0.052072937

## Discussion

In this study, we analyzed the HIV-1 pol gene sequences of 223 ART treatment-naïve MSM in Beijing diagnosed from 2011 to 2013. The genotype CRF01_AE accounted for 60.5%, followed by CRF07_BC (27.8%), subtype B (9.9%), and others. Fifty-seven samples had at least one TDR-associated mutation, mainly including L10I/V (6.3%), A71L/T/V (6.3%), V179D/E (5.4%), and V106I (2.7%), with different distributions of TDR-associated mutations by different HIV-1 subtypes and by sample year. Moreover, eight significant co-variation pairs were found between TDR-associated mutations (V179D/E) and seven overlapping polymorphisms in subtype CRF01_AE. This analysis consists the most comprehensive genetic characterization to date on HIV-1 TDR associated mutations prevalent among MSM in China.

The proportion of samples with TDR-associated mutations over the sample period was determined to be 25.6%. The mutations frequency rose from 14.3% in 2011 to 29.4% in 2012 and 22.4% in 2013. In addition, the distribution of mutations sites in 2011 was simpler compared with 2012 and 2013. This indicated that TDR-associated mutations may be becoming increasingly common and complex among Beijing MSM over the last several years. Although most of these mutations only conferred potential drug resistance, TDR-associated mutations often confer disadvantage in replicative fitness to the virus. A high prevalence and increasingly complex patterns of TDR-associated mutations suggests a generally high frequency of exposure in the treatment-naïve patients to diverse sources of viral strains. Thus, TDR-associated mutations in Beijing MSM may serve as evidence for prevalent high risk behavior such as repeated unprotected exposure to multiple sexual partners.

The largest proportion of the HIV-1 strains among our samples belonged to the CRF01_AE genotype (60.5%), followed by CRF07_BC (27.8%) and subtype B (9.9%), and the distribution of HIV-1 subtypes was relatively stable from 2011 to 2013. This differed from previous reports on Beijing MSM from 2005, which saw higher prevalence of subtype B and lower prevalence of CRF01_AE and CRF07_BC [16]. In the study performed by Li et al. from 2007 to 2010 [17], the proportion of subtype CRF01_AE, B, and CRF07_BC were 56.0%, 30.8%, and 12.6%, respectively. Comparing to previous reports, the ratio of subtype B shows a tendency to drop, while the proportions of CRF01_AE and CRF07_BC are continuously on the rise in MSM population in Beijing. Historically, CRF01_AE was strongly associated with sexual transmission routes [23], while CRF07_BC was associated with intravenous drug user populations in China [24],[25]. It is likely that the increase in CRF07_BC prevalence among MSM was linked with transmission among drug users [26].

We found unequal distributions of mutations in the different genotypes, with subtype B having a higher rate (54.5%) of TDR-associated mutations than in CRF07_BC and CRF01_AE. This observation coincides with results from a study among Thai patients, in which multivariate analysis showed that HIV-1 subtype B had a higher rate of drug resistance-associated mutations [27]. On the other hand, TDR-associated mutations among CRF01_AE strains showed a diversifying trend. Nonetheless, the most frequent mutations L10I/V and V179D/E appeared to be conserved; the latter was found in 9 out of 11 CRF01_AE strains with drug resistance mutations to NNRTI drugs.

The relative prevalence of V179D/E bears further remark. In a previous study, Archer et al. found that V179D (a NNRTI resistance mutation) significantly reduced the replication capacity of HIV-1 [28]. Here, our co-variation analysis showed that V179D/E was significantly associated with seven polymorphisms in the HIV-1 CRF01_AE genotype. These polymorphisms may serve to compensate the replication disadvantage of V179D/E, allowing this TDR-associated mutation to be propagated in treatment-naïve patients [5]. In another study by our team, we had applied the CorMut algorithm to investigate the association between drug resistance and compensatory mutations, and demonstrated that K101Q, H221Y, and T139K can enhance K103N/Y181C/G190A-associated NNRTI-resistance among CRF07_BC in vitro [29]. Certainly, further study is needed to determine how the V179D/E-associated polymorphisms affect the replication fitness of CRF01_AE HIV-1 strains.

However, there are some insufficiency still exists in our study. First, the study patients were newly diagnosed, but we can’t confirm the time of initial infection. Some TDR-associated mutations have been missing during this period. Another limitation of the study is related to the use of population sequencing strategy. Such a conventional genotyping technique does not allow for the detection of clinically important minority variants with TDR-associated mutations [30]. Despite some limitations, current study may facilitate the tracking of TDR-associated mutations, and should provide a reliable data on prevalence of the TDR-associated mutations among MSM in Beijing.

## Conclusions

Our work comprehensively characterized HIV-1 strains prevalent among treatment-naïve MSM in Beijing from 2011 to 2013, including subtype analysis, TDR-associated mutations, and co-variation of potential compensatory mutations. TDR mutation rate remains low, but the rate of TDR-associated mutations is high. There are significant co-variation pairs between TDR-associated mutations (V179D/E) and seven overlapping polymorphisms among subtype CRF01_AE. These findings enhance our understanding of TDR and evolution of HIV-1 among Chinese MSM, and suggest that high risk behavior patterns that facilitate the transmission of drug resistant HIV strains remain highly prevalent, requiring stronger prevention and control efforts.

## Authors’ original submitted files for images

Below are the links to the authors’ original submitted files for images. Authors’ original file for figure 1
