# Supplementary figures and images for: HIV-1 transmitted drug resistance-associated mutations and mutation co-variation in HIV-1 treatment-naïve MSM from 2011 to 2013 in Beijing, China
Source: BMC Infect Dis. 2014 Dec 16;14:689. doi: 10.1186/s12879-014-0689-7 (PMC4271504; doi:10.1186/s12879-014-0689-7)

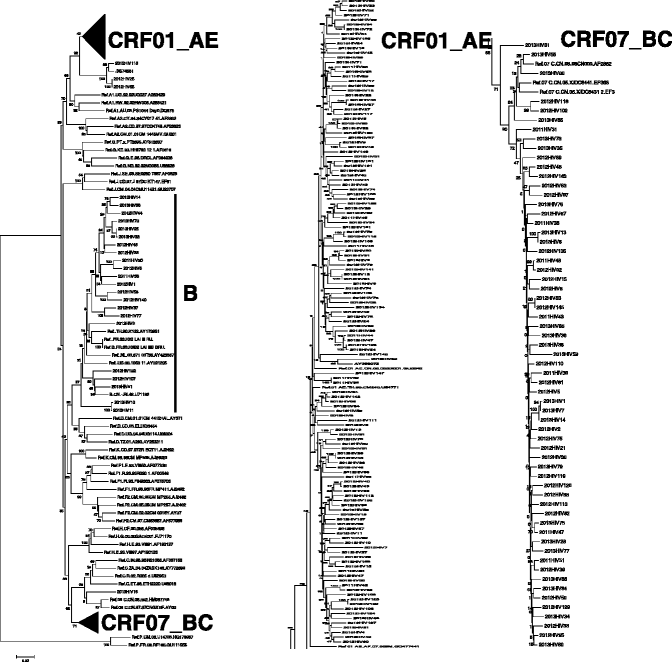

Supplement: Supplementary file 1 — Authors’ original file for figure 1 [file 12879_2014_689_MOESM1_ESM.gif]
